# Deterministic Domain Wall Motion Orthogonal To Current Flow Due To Spin Orbit Torque

**DOI:** 10.1038/srep11823

**Published:** 2015-07-03

**Authors:** Debanjan Bhowmik, Mark E. Nowakowski, Long You, OukJae Lee, David Keating, Mark Wong, Jeffrey Bokor, Sayeef Salahuddin

**Affiliations:** 1 Department of Electrical Engineering and Computer Sciences, University of California Berkeley, Berkeley, CA 94720, USA; 2 Department of Physics, University of California Berkeley, Berkeley, CA 94720, USA; 3 Material Science Division, Lawrence Berkeley National Laboratory

## Abstract

Spin-polarized electrons can move a ferromagnetic domain wall through the transfer of spin angular momentum when current flows in a magnetic nanowire. Such current induced control of a domain wall is of significant interest due to its potential application for low power ultra high-density data storage. In previous reports, it has been observed that the motion of the domain wall always happens parallel to the current flow – either in the same or opposite direction depending on the specific nature of the interaction. In contrast, here we demonstrate deterministic control of a ferromagnetic domain wall orthogonal to current flow by exploiting the spin orbit torque in a perpendicularly polarized Ta/CoFeB/MgO heterostructure in presence of an in-plane magnetic field. Reversing the polarity of either the current flow or the in-plane field is found to reverse the direction of the domain wall motion. Notably, such orthogonal motion with respect to current flow is not possible from traditional spin transfer torque driven domain wall propagation even in presence of an external magnetic field. Therefore the domain wall motion happens purely due to spin orbit torque. These results represent a completely new degree of freedom in current induced control of a ferromagnetic domain wall.

According to the conventional spin transfer torque model, when spin polarized electrons coming from a magnetized region impinge on the spins in a domain wall, they exert a torque that tries to orient the domain wall spins in the direction of the incoming spins^1^,^2^,^3^,^4^. The strength of the torque is proportional to the relative angle between the incoming spins and domain wall spins. If a domain wall is formed along the width of a perpendicularly polarized magnetic wire (transverse domain wall) as shown in Fig. 1a, then a current flowing in the long direction can exert such a torque and move the domain wall. This has been the canonical configuration for studying current induced domain wall motion^1^. If, on the other hand, a domain wall is formed along the length as shown in Fig. 1b, d, then no conventional spin transfer torque, to be referred to as the bulk spin torque, is exerted on this longitudinal wall by a current that flows along the length (longitudinal current), even in the presence of an external in-plane magnetic field (see Section S1 of Supplementary Information for micromagnetic simulation showing this effect). This is because the domain wall spins do not change direction along the path of the current in this case. In this paper, by exploiting the new phenomenon of current induced spin accumulation using the spin-orbit interaction in a ferromagnet-heavy metal heterostructure^5^,^6^,^7^,^8^,^9^,^10^,^11^,^12^,^13^,^14^,^15^,^16^,^17^,^18^,^19^,^20^, we show that, indeed, a longitudinal current can deterministically move a longitudinal domain wall in the presence of a longitudinal magnetic field. Reversing the direction of the current flow or the direction of the field reverses the direction of the domain wall motion. This also presents a unique insight into the spin-orbit torque driven switching of perpendicularly polarized magnets.

Recently, spin orbit torque (SOT) has been used by Ryu *et al.*^6^ and Emori *et al.*^7^ to move domain walls in a narrow wire using a current flow. Both these works show how a transverse domain wall, created in a heavy metal/ ferromagnet heterostructure, can be moved by a current flow due to a simultaneous action of spin orbit torque and Dzyaloshinskii-Moriya Interaction (DMI) at the heavy metal/ ferromagnet interface. Nonetheless, the structure of the domain wall used in these experiments is transverse which is identical to the conventional structure where a bulk spin torque could also move the wall along the direction of current flow. By contrast, we have created a longitudinal domain wall and have shown that such a wall, which cannot be moved by bulk spin torque, can be deterministically moved by the spin orbit torque orthogonal to the current flow. This is enabled by the fact that the current flowing through the structure leads to a spin accumulation at the heavy metal (Ta) / ferromagnet (CoFeB) interface, which is orthogonal to the current direction (Fig. 1(c))^12^. This is different from the case of bulk spin torque where the spins impinging on the magnetic domain wall are collinear with the source magnetization. Thus our work shows that the otherwise well-known spin orbit torque resulting from a realtive mis-alignment of the magnetization and spin acuumulation can be exploited in a novel geometry to demonstrate a new type of domain wall motion orthogonal to the current flow.

## Results

### Creation of a longitudinal ferromagnetic domain wall and its motion under an out of plane magnetic field

**Figure 1:**
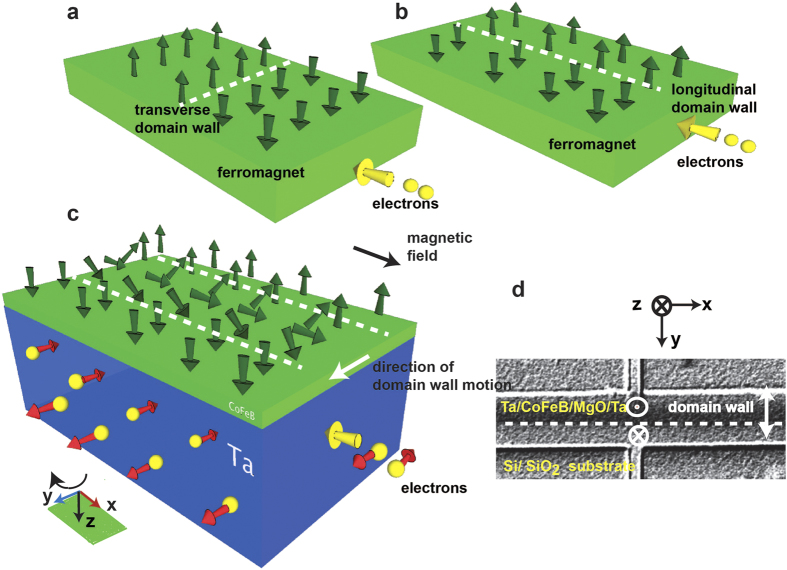
Spin orbit torque driven domain wall motion. **a,** Current flows across a transverse domain wall. Green arrows represent the magnetic moments. **b,** Current flows along a longitudinal domain wall. **c,** When current flows through the Ta in x direction (electrons in –x direction) electrons with spin polarization in -y direction accumulate at the interface of the Ta and CoFeB. This results in the transfer of spin orbit torque to the domain wall in CoFeB. The green parallelogram represents the plane of the sample and the three arrows show the directions of x, y and z-axes. The z-axis points in the direction of the vector product of x and y axes following the right hand rule, which is into the plane (⊗). **d,** The MOKE image of the 20 microns wide magnetic bar shows the formation of a longitudinal wall along it when current flows in the +x direction. The longitudinal domain wall at y = 0 separates an “out of the plane” (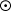) polarized domain for y <0 and an “into the plane” (⊗) polarized domain for y > 0. Anomalous Hall voltage is measured across the orthogonal bar.

**Figure 2:**
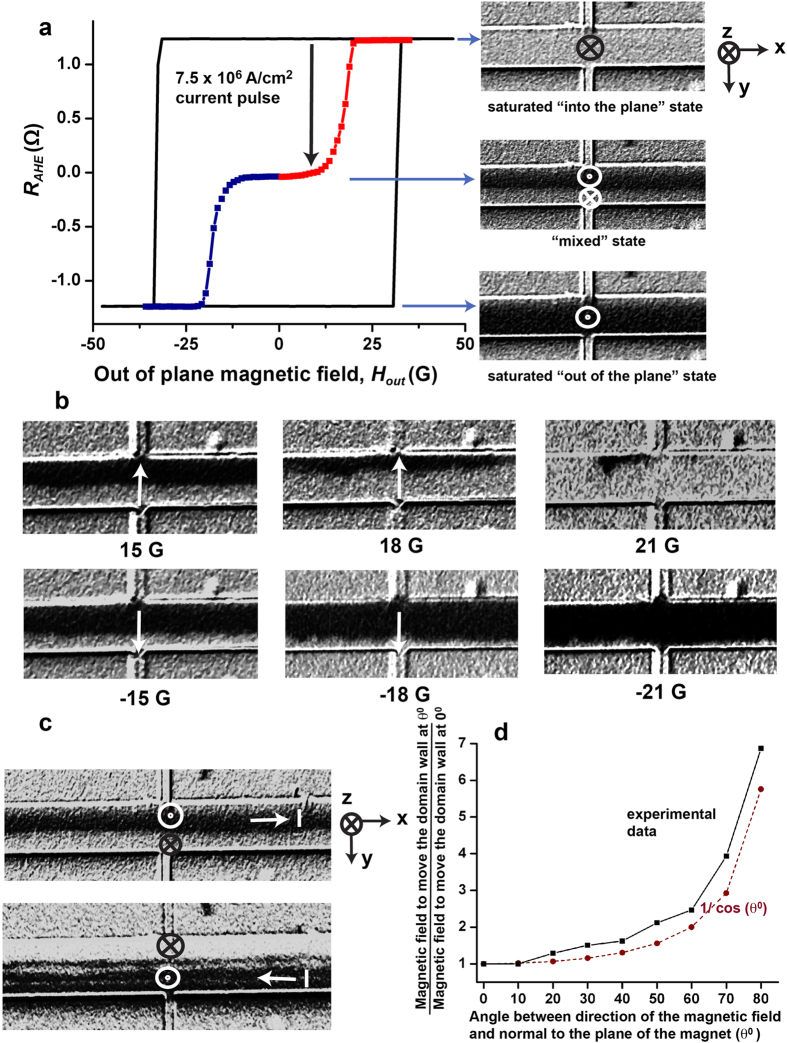
Creation and magnetic field based control of the longitudinal domain wall. **a,** Anomalous Hall resistance (R_AHE_) measurement shows that the magnet can be switched between a saturated “into the plane” (⊗) polarized state (m_z_ = 1 or R_AHE_ = 1.2 Ω) and a saturated “out of the plane” (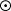) polarized state (m_z_ = −1 or R_AHE_ = −1.2 Ω) with a switching field of ~30 gauss. Starting from the m_z_ = 1 state, a current pulse of magnitude 7.5 × 10^6^ A/cm^2^ is applied along the bar in +x direction at a zero magnetic field. At the end of the pulse R_AHE_ is measured to be 0 Ω. This corresponds to the “mixed state” of the bar. MOKE image of the “mixed state” shows a longitudinal domain wall with a m_z_ = −1 polarized domain for y < 0 and a m_z_ = 1 polarized domain for y > 0. When out of plane magnetic field is applied in z direction on the “mixed” state, R_AHE_ does not change till ~15 G. Beyond that, R_AHE_ increases till the magnet reaches the saturated “into the plane” state (m_z_ = 1, R_AHE_ = 1.2 Ω) - red plot. Applying another current pulse of magnitude 7.5 × 10^6^ A/cm^2^ on the saturated state creates the “mixed state” with a longitudinal domain wall again. Now applying a magnetic field in -z direction causes reduction in R_AHE_ till the magnet gets saturated in the “out of plane” direction (m_z_ = −1, R_AHE_ = −1.2 Ω) - blue plot. **b,** MOKE images of the magnetic bar under the application of “into the plane” (+z) field shows the movement of the longitudinal domain wall in -y direction so that the average magnetization is aligned with the field. When “out of the plane” (-z) field is applied, the longitudinal domain wall moves in +y direction. **c,** Mixed state, formed by a positive current pulse (current I along +x) has “out of the plane” (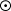) polarized domains for y < 0 and “into the plane” (⊗) polarized domains for y > 0 whereas mixed state, formed by a negative current pulse (I along −x) has “into the plane” (⊗) polarized domains for y < 0 and “out of the plane” (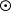) polarized domains for y > 0. **d,** Minimum field needed to move the longitudinal domain wall from the centre of the bar is plotted against the angle of application of the field.

For experimental investigation, Hall bars are fabricated from a stack of Si (substrate)/ SiO_2_ (100 nm)/ Ta (10 nm)/ CoFeB (1 nm)/ MgO (1 nm)/ Ta (2 nm), the CoFeB layer being the ferromagnet and the bottom Ta layer of thickness 10 nm is the heavy metal which provides the spin orbit torque. The thickness of the CoFeB layer is chosen to be 1 nm and a layer of MgO is deposited on the top of it to ensure that the CoFeB layer exhibits perpendicular magnetic anisotropy^10^ (Section S2 of Supplementary Information). The upper layer of Ta with thickness of 2 nm is used as a capping layer for the stack. Current is applied along the bar of width 20 microns, which is along the x-axis, and anomalous Hall voltage is measured in the y direction across the narrower bar of width 5 microns (Fig. 1d). The anomalous Hall voltage is proportional to the average out of plane magnetization at the intersection region of the two bars^11^,^12^. Anomalous Hall resistance (R_AHE_) is the ratio of the anomalous Hall voltage to the small constant current applied to measure the voltage ( 5 × 10^4^ A/cm^2^ for all our measurements). The anomalous Hall resistance (R_AHE_) versus magnetic field plot of Fig. 2a shows that the CoFeB layer exhibits perpendicular anisotropy with a magnetic field of ~30 G needed to switch its magnetization between the two saturated states- “into the plane” or m_z_ = 1 state (⊗) with R_AHE_ = 1.2 Ω and “out of the plane” or m_z_ = −1 state (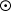) with R_AHE_ = −1.2 Ω; m_z_ is the magnetization in z-direction, normalized by the saturation magnetization. We follow the following convention consistently throughout the paper: x and y axes are two orthogonal directions on the plane of the sample and the cross product of x axis and y axis is z axis (x × y), following right hand rule. The axes are chosen such the positive z axis is “into the plane” and negative z axis is “out of the plane” (Figs 1c,d and Fig. 2a).

Along with measurement of R_AHE_, Magneto Optic Kerr Effect (MOKE) has been used to image the magnetic domains of the wider Hall bar^6^,^7^,^8^. To obtain the best contrast, each image has been subtracted from a reference image of a saturated “into the plane” state of the magnet. The subtracted image of the saturated “into the plane” state of the magnet shows no contrast between the magnetic bar and the background substrate material because the substrate does not contribute to a magnetic signal and the positive magnetic signal of the “into the plane” magnet when subtracted from another image of “into the plane” magnet yields zero (Fig. 2a). On the other hand, when an image of a saturated “out of the plane” state is subtracted from the reference image of “into the plane” state, the substrate still gives no signal, but the difference between negative signal of the “out of the plane” magnet and positive signal of the “into the plane” magnet is non-zero. So a subtracted image of “out of the plane” state shows a dark contrast between the magnetic bar and the background (Fig. 2b).

Starting from the magnet saturated “into the plane” (m_z_ = 1, R_AHE_ = 1.2 Ω), a current pulse of magnitude 7.5 × 10^6^ A/cm^2^ and duration 1 s is applied along the wider Hall bar in +x direction at a zero magnetic field (Fig. 1d). At the end of the pulse, the steady state R_AHE_ of the final magnetic state is measured to be ~0 Ω (Fig. 2a). The MOKE image of that state shows the magnet to be split into a domain of “out of the plane” or m_z_ = −1 (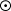) polarized moments for y < 0 and a domain of “into the plane” or m_z_ = 1 (⊗) polarized moments for y > 0 with a longitudinal domain wall separating the two, based on the chosen coordinate system (Fig. 2a and 1d)^21^. We call this state the “mixed” state (Fig. 2a) because the magnet has both m_z_ = 1 and m_z_ = −1 polarized domains in this state. Reversing the polarity of the current reverses the position of the two domains in the “mixed” state (Fig. 2c). The polarity of the domains in the “mixed” state follows the out of the plane component of the Oersted field, generated by the current pulse, at the edges of the bar. Micromagnetic simulations in Section S3 of Supplementary Information show that starting from a saturated state, reverse polarized domains are nucleated at the edge of the bar due to the Oersted field. Subsequently, the domain wall moves from the edge of the bar to the centre to reduce the magnetostatic energy of the system. Once the longitudinal domain wall is formed at the centre of the bar after the application of the current pulse, application of an out of plane magnetic field moves the domain wall in one direction or another based on the polarity of the field as observed from the MOKE images (Fig. 2b). The displacement of the domain wall is proportional to the magnitude of the applied field, indicating that the domain wall motion is governed by pinning defects^22^,^23^. We also observe that the domain wall starts moving from the centre of the bar only when the out of plane field is 15 G or above. If the magnetic field is next applied at different angles (θ) with respect to the film normal, the magnetic field needed to move the domain wall follows a 1/cos(θ) dependence as expected from the Kondorsky model of domain wall depinning field^22^ (Fig. 2d).

### Motion of the longitudinal domain wall orthogonal to current flow in presence of an in-plane magnetic field

**Figure 3:**
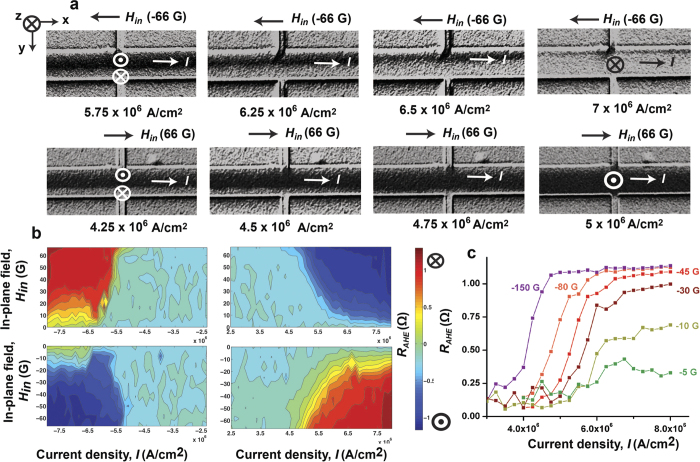
Movement of longitudinal domain wall orthogonal to the current. **a,** MOKE images of the magnetic bar after a positive current pulse of different magnitude is applied on a longitudinal domain wall (“mixed” state of Fig. 2a) in the presence of an in-plane field (*H*_*in*_) in –x and +x direction. **b,** Contour plots of final R_AHE_ after application of a current pulse versus the magnitude of the current and magnitude of the applied in-plane field for different combinations of positive and negative currents and fields. **c,** Plots of R_AHE_ versus magnitude of current pulse for different values of magnetic field, applied in –x direction.

Creation of such a longitudinal domain wall provides us with the unique opportunity to control its motion orthogonal to the current flow using spin orbit torque, which is not otherwise possible from a bulk spin torque. Deterministic control of the motion of this domain wall orthogonal to the current flow is the central point of the paper, which we discuss next. We first saturate the magnetic bar in “into the plane” (m_z_ = 1) state and apply a current pulse of magnitude 7.5 × 10^6^ A/cm^2^ and duration 1 s along the bar in +x direction at zero magnetic field to create a “mixed” state with a longitudinal domain wall at the center of the bar just as in Fig. 2a. Starting from the longitudinal domain wall, a current pulse of duration 1 s is applied along the bar in the +x direction in the presence of an in-plane magnetic field in the –x direction (Fig. 3a). This is repeated for different magnitudes of the current pulse. MOKE images of the bar taken after every current pulse show that the current pulse moves the domain wall in -y direction such that the “into the plane” or m_z_ = 1 polarized domain expands while the “out of the plane” or m_z_ = −1 polarized domain contracts. The distance moved by the domain wall is proportional to the magnitude of the current pulse. Reversing the direction of the magnetic field reverses the direction of the domain wall motion because when the magnetic field is in +x direction and the current is also in +x direction, the domain wall moves in the +y direction and the final state of the magnet is in –z direction (Fig. 3a). We next vary the in-plane magnetic field and apply current pulses of different magnitude starting every time from the same initial condition of a longitudinal domain wall at the centre of the bar just as in “mixed state” of Fig. 2(a). We measure R_AHE_, indicative of the domain wall position, after each current pulse and plot the R_AHE_ as a function of the current and the in-plane field. The contour plot obtained in that process [Fig. 3(b)] shows that when the in-plane magnetic field is positive (along +x) current along +x (positive polarity) beyond a threshold value (~5 × 10^6^ A/cm^2^) moves the domain wall in +y direction and hence the average m_z_ < 0 (R_AHE_ < 0, blue colour in contour plot). The higher the current or stronger the field, the more negative is the average m_z_ and the R_AHE_. In the presence of a positive in-plane magnetic field (along +x), current in −x direction (negative polarity) moves the domain wall in -y direction and the average m_z_ > 0 (R_AHE_ > 0, red colour in contour plot). When in-plane magnetic field is applied in –x direction (negative field), positive current pulse moves the domain wall in -y direction (R_AHE_ > 0, red colour) and negative current pulse moves the domain wall in +y direction (R_AHE_ < 0, blue colour). The R_AHE_ measurement results of Fig. 3b are consistent with the MOKE images of Fig. 3a in terms of the direction of domain wall motion and final state of the magnet under different combinations of current pulse and in-plane magnetic field (see Section S4 of Supplementary Information for MOKE images of domain wall motion under a negative current pulse, which are also consistent with R_AHE_ measurement of Fig. 3b).

The final state of the magnet under different combinations of polarities of applied current and applied in-plane magnetic field is consistent with the magnetic switching experiment we performed on our devices, starting from a saturated magnetic state, (Section S9 of Supplementary Information) as well as that performed by Liu *et al.*^12^ Such hysteric switching of the magnet with current in the presence of in-plane magnetic field is a signature of spin orbit torque at the Ta-CoFeB interface.

### Micromagnetic simulations showing spin orbit torque driven motion of the longitudinal domain wall

**Figure 4:**
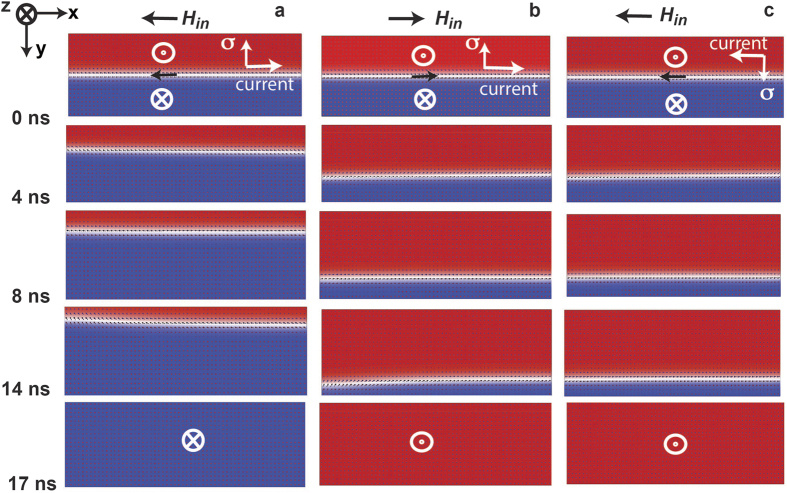
Micromagnetic simulations of domain wall motion orthogonal to the current. **a, b,** Micromagnetic simulation of a 600 nm long and 200 nm wide magnet shows the motion of a longitudinal domain wall under the application of a spin polarization 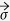 in –y direction (current *I* in +x direction) and an in-plane magnetic field (*H*_*in*_) in –x/ +x direction. The direction of the net magnetic moment in the domain wall, shown by a black arrow, is initialized along the direction of applied field. **c,** Micromagnetic simulation of the same magnet with spin polarization in +y direction (current I in –x direction) and magnetic field in –x direction.

We performed micromagnetic simulations in Object Oriented Micromagnetic Framework to explain this result^24^,^25^. Starting from a longitudinal domain wall at the centre of a rectangular magnet of length 600 nm, width 200 nm and thickness 1 nm, the system is allowed to evolve with time under the influence of an in-plane magnetic field along its length and a Slonczewski-like spin orbit torque^7^,^12^,^26^ orthogonal to it. Current flowing through a heavy metal-ferromagnet heterostructure is known to apply a Slonczweski-like spin orbit torque on the ferromagnet of the form 
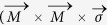
) where 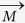 is the magnetization of the ferromagnet and 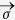 is the direction of spin polarization of the accumulated electrons at the heavy metal-ferromagnet interface, which is determined by the direction of the current^7^,^12^,^26^. We consider the longitudinal wall to be a Bloch wall. Both Bloch walls and Neel walls have been observed in thin films exhibiting perpendicular magnetic anisotropy in previous studies^7^,^8^,^27^,^28^. Dzyaloshinskii-Moriiya Interaction (DMI) is known to stabilize Neel walls of a particular chirality in thin films^7^,^27^. Emori *et al.*^18^ and Torrejon *et al.*^29^ have reported that the strength of the DMI is very low in Ta/CoFe/MgO and Ta/CoFeB/MgO heterostructures respectively. The DMI is also found to be negligible in our samples (Section S5 of Supplementary Information). Also, in the case of the longitudinal domain wall, which we study here, magnetostatic energy is lowered when the moments inside the domain wall are oriented along the length of the wall owing to the shape anisotropy. Thus the longitudinal domain wall formed in our system is likely to be a Bloch wall with moments inside the wall oriented along its length (Fig. 1c). In addition, we observe the current driven domain wall motion in the presence of an external magnetic field, applied in-plane along the length of the wall (Fig. 3 and 4). In the presence of this field, the moments inside the wall prefer to orient along the direction of the field (Fig. 1c)^18^,^27^. Micromagnetic simulations (see Methods for details of the method of simulation) show that when a magnetic field is applied in –x direction and a current is applied in the +x direction the domain wall moves in –y direction till the magnet is saturated into the plane (Fig. 4a). Reversing the direction of the magnetic field (Fig. 4b) or the current (Fig. 4c) reverses the direction of domain wall motion, just as observed experimentally (Fig. 3). Thus, polarity of the final state of the magnet is found to be a cross product of direction of the in-plane field and direction of accumulated spins. Notably, these simulations results, which show a one-to-one correspondence with the experimental observation, are consistent with the Slonczewski-like spin orbit torque. Oersted field or field like spin orbit torque from the current cannot explain such domain wall motion^13^,^26^ (Section S6 of Supplementary Information). Since the direction of the domain wall motion depends on the direction of the current and the in-plane magnetic field, Joule heating due to the current cannot explain the result. However we measured the temperature change of our device due to Joule heating. For the current magnitudes used, temperature of the device can go up to 400 K starting from room temperature (Section S10 of Supplementary Information).

## Discussion

An intuitive explanation for our experimental and simulation results can be obtained by analyzing the Landau Lifschitz Gilbert equation, which determines the magnetization dynamics, modified by two spin orbit torque terms- Slonczweski type torque of the form 
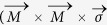
) and field like torque of the form 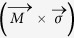
26:
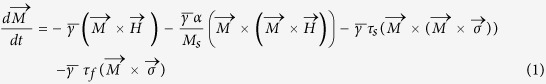
where 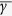 is the gyromagnetic ratio, *α* is the damping constant, *τ*_*s*_ is the Slonczweski spin orbit torque coefficient and *τ*_*f*_ is the field-like torque coefficient. We have found experimentally that our longitudinal domain wall does not move under the application of an in-plane field alone even if the field is as high as 500 G (Section S8 of Supplementary Information). In the field-like torque term of the equation (1) : 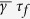 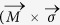), 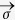 acts like an in-plane field. Hence the field-like torque cannot move the domain wall. If the longitudinal domain wall is a Bloch wall, 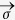 and the average magnetization of the domain wall 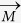 are orthogonal and 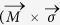) can act as an effective magnetic field (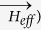 which can move the domain wall [
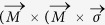
))=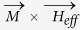]^7^,^23^. In the absence of an external magnetic field, parts of the domain wall will have magnetization in one direction and other parts will have it in other direction. The average 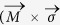) experienced by the wall is 0 and so the wall does not move. On the other hand, under the application of the magnetic field along the domain wall, all the moments inside the wall align along the direction of the field and the wall experiences a non-zero effective magnetic field which moves the domain wall. When the magnetic field is along –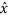 (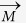
along –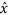) and current is along 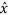 (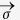
along –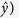
the effective out of plane field experienced by the domain wall (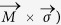 is in +z direction (“into the plane”) and the domain wall moves in –y direction so that final state of the magnet is in +z direction (Fig. 3 and 4). When magnetic field is along 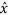 and current is along 
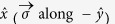
or when magnetic field is along –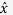
and current is along –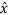 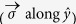, effective field (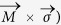 is in –z direction. So the domain wall moves in +y direction so that the final state of the magnet is in –z direction (“out of the plane”) (Fig. 3 and 4).

One way to make a quantitative estimate for the efficiency of the spin-orbit torque is to relate the current density needed to move the domain walls to the effective out-of plane field, following Thiaville *et al.*^30^ Starting from the same initial condition of a longitudinal wall at the centre of the magnetic bar identical to the “mixed state” of Fig. 2(a), current pulses of different magnitude are applied along the bar in the +x direction with a magnetic field applied in –x direction (Fig. 3). R_AHE_ is measured after each pulse to indicate the position of the domain wall. The same experiment is repeated for different values of in-plane magnetic field. We observe that for very small in-plane fields current pulses up to the magnitude of 8 × 10^6^ A/cm^2^ move the domain wall barely (Fig. 3c). For an in-plane magnetic field of magnitude 45 G and above, the distance moved by the domain wall, is linearly proportional to the current density (J_c_) of the applied pulse. As a result the R_AHE_ measured after the current pulse varies linearly with current density till the domain wall moves to the edge of the bar to switch the entire magnet and hence R_AHE_ reaches saturation. The slopes of the linear regions of the curves (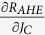) are approximately the same for different magnitudes of in-plane magnetic field above 45 G and the average slope is equal to 7.39 × 10^−7^ Ω/(A/cm^2^) (see Section S7 of Supplementary Information for details of the calculation). Fig. 2a shows that the longitudinal domain wall moves under the application of an out of plane field such that the R_AHE_ varies with the out of plane field at a rate of 0.15 Ω/G (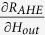), obtained by linear fitting of the curve (see Section S7 of Supplementary Information). Comparing the two slopes, we conclude that for an in-plane field sufficient enough to switch the net magnetic moment in the domain wall to the direction of the field such that the moment is orthogonal to the direction of spin polarization of the accumulated electrons^7^,^30^,^31^,^32^ the current (J_c_) acts as an effective out of plane field (H_out_) such that 
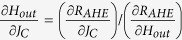
 = 4.92 × 10^−6^ G/(A/cm^2^). We can relate this slope to the expression 
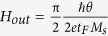
30, where θ is the efficiency of spin orbit torque, e is the charge of an electron,*ħ* is the Planck constant, M_s_ is the saturated magnetization of the ferromagnet (800 e.m.u/c.c.), as measured for our thin film stack by Vibrating Sample Magnetometry) and t_F_ is the thickness of the ferromagnet (1 nm). This provides θ = 0.076. The value of the spin orbit torque efficiency is expected to depend on the growth conditions, which determine the overall quality of the materials and interfaces. For example, for the similar stack (Ta/CoFeB/MgO) as ours, spin Hall angle values for Ta ranging from 0.02^33^ to 0.12^12^ have been reported.

To summarize, we have shown that spin orbit torque can be exploited to deterministically move a domain wall in a perpendicularly polarized magnet orthogonal to current flow, which is otherwise not achievable by conventional bulk spin torque. Reversing the polarity of the current or the in-plane magnetic field reverses the direction of motion of the domain wall. This adds a new capability to the toolset of current induced domain wall control and can impact the way a domain wall is routed through complicated structures^34^,^35^. The specific configuration presented in this work also provides a way to investigate the spin-orbit torque directly, unaffected by any bulk spin torque which is zero by symmetry.

## Methods

### Sample preparation

A thin film stack of Ta (10 nm)/ CoFeB (1 nm)/MgO (1 nm)/ Ta (2 nm) has been sputter deposited on thermally oxidized Si substrate at room temperature. Vibrating Sample Magnetometry is used to characterize the magnetic properties of the thin film stack and ensure that the stack exhibits perpendicular magnetic anisotropy (Section S2 of Supplementary Information). Orthogonal Hall bars are fabricated from it using optical lithography and ion milling. The bar along x-axis (Fig. 1d) is 500 μm long and 20 μm wide and the bar along y-axis is 500 μm long and 5 μm wide.

### Anomalous Hall resistance measurement

Anomalous Hall resistance (R_AHE_) is measured by applying dc current of 100 μA (current density- 5 × 10^4^ A/cm^2^) along the bar along the x-axis and measuring the Hall voltage across the bar along y-axis with a nanovoltmeter (Fig. 1d). The current applied for R_AHE_ measurement is 2 orders of magnitude lower than that used for creating the “mixed” state or moving the domain walls.

### Magneto-optic Kerr effect imaging

A Magneto-Optic Kerr Effect (MOKE) microscope is used for magnetic imaging of the Hall bars. The MOKE microscope consists of a 455 nm LED source, two polarizers and a 0.45 NA objective, nominally giving a resolution of 1 μm. To observe contrast, first the magnet is saturated in the “into the plane” or +z direction and an image is taken (Fig. 2a). This is our reference image. Then a current pulse, a magnetic field or both are applied and another image is taken. Then the two images are aligned and the reference image is subtracted from the other to generate the final MOKE image, which we use for this work. When the bar is saturated “into the plane” the background substrate does not contribute to magnetic signal but the bar does. So when the reference image of an “into the plane” saturated magnet is subtracted from another image of “into the plane” magnet, the final image shows no contrast between the bar and the background (Fig. 2a- “into the plane” (⊗) saturated magnet). When the bar is saturated in the “out of plane” direction, there is no signal from the background substrate but the signal from the bar is strong and negative of the signal from the bar when it is saturated in the “into the plane” direction. As a result when an image of “into the plane” saturated magnet, which is the reference image, is subtracted from the image of “out of the plane” saturated magnet, the resulting MOKE image shows a dark contrast between the bar and the background substrate (Fig. 2a-“out of the plane” (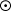) saturated magnet).

### Pulsing experiment

Each data point in the plots of Fig. 3b,3c of the main paper is obtained by first saturating the magnet to “into the plane” (m_z_ = 1) state, then applying a current pulse of magnitude 7.5 × 10^6^ A/cm^2^ and duration 1 s along the bar in +x direction at zero magnetic field to create the longitudinal domain wall and finally applying another current pulse of a certain magnitude and polarity at a certain in-plane magnetic field. At the end of the pulse anomalous Hall resistance (R_AHE_) is measured and plotted against the current applied with the second pulse and the in-plane magnetic field applied. All the data in this letter are obtained with current pulses of duration 1 s. For the current range used to move the longitudinal domain wall, which is between 4 × 10^6^ A/cm^2^ and 7 × 10^6^ A/cm^2^ (Fig. 3), the rise time of the current pulse is between 20 and 30 μs. The applied in-plane magnetic field is dc and is present throughout the duration of the domain wall movement from centre of the bar to the edge (Fig. 3). All the measurements are performed at room temperature.

### Simulation

Micromagnetic simulations are performed in Object Oriented Micromagnetic Framework (OOMMF)^24^ using the spin torque extension module- CYY_STTEvolve^25^. A 600 nm long, 200 nm wide and 1 nm thick magnet is simulated with a 2 nm mesh size laterally and 1 nm mesh size along the thickness (Fig. 4). Thus the simulation is basically performed on a two dimensional grid. At every point in the grid (x, y), the moment is allowed to evolve under time following the Landau Lifschitz Gilbert equation with the Slonczewski spin transfer torque term:
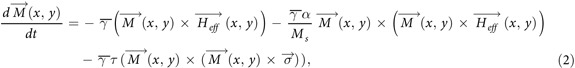
where 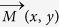 is the magnetization at a point in the grid with coordinates (x,y) (Fig. 4) and can point in any direction in the (x,y,z) space, 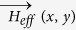 is the effective field experienced by the magnetization at that point (x,y), α- damping constant, 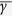 - gyromagnetic ratio, 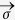
- direction of spin polarization and 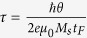
11, θ- spin orbit torque efficiency, J_c_- charge current, e- charge of an electron, μ_0_- vacuum permeability, M_s_- saturation magnetization of the ferromagnet (8 × 10^5^ A/m or 800 e.m.u./c.c. used for simulation) and t_F_- thickness of the ferromagnet ( 1 nm).

The effective field 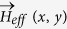 is calculated using 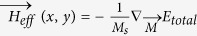, where 

the total energy density E_total_=E_anisotropy_+E_exchange_+E_Zeeman_+E_magnetostatic_

Anisotropy energy E_anisotropy_ = −K M_z_^2^, corresponding to the perpendicular magnetic anisotropy of the ferromagnet. K = 6 × 10^5^ J m^–3^ = 6 × 10^6^ erg/c.c. is used in the simulation.

Exchange energy E_exchange_ = *A*  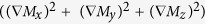, where A is the exchange correlation constant (3 × 10^−11^ J m^−1^ or 3 × 10^−6^ erg cm^−1^ ).

Zeeman energy E_Zeeman_ = - 
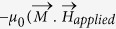
) where 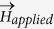 is the applied magnetic field.

E_magnetostatic_ is the magnetostatic energy or dipole energy of the system, calculated by the micromagnetic simulator.

The initial condition of the simulations in Fig. 4 is a longitudinal domain wall, which divides the magnet into oppositely polarized domains. To simulate that we use the function M_z_ (x,y)= - M_s_ cos(Ψ); M_x_ (x,y)= M_s_ sin(Ψ) for H_applied,x_ > 0 and M_x_ (x,y) = −M_s_ sin(Ψ) for H_applied,x_ < 0, where 
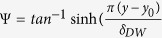
 )^36^, y_0_-position of the domain wall, which is the centre of the bar initially (t=0 s) and δ_DW_= domain wall width = 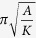 = 22 nm.

The system is allowed to evolve using equation (1) under the application of a 10 G magnetic field.

(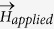) in +x/−x direction and a spin polarization of 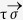 where 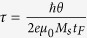 and 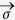 = -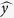 (Fig. 4) with θ = 0.08, M_s_ = 8 × 10^5^ A/m = 800 e.m.u./c.c., t_F_ = 1 nm and J_c_ = 3.5 × 10^6^ A/cm^2^.

## Additional Information

**How to cite this article**: Bhowmik, D. *et al.* Deterministic Domain Wall Motion Orthogonal To Current Flow Due To Spin Orbit Torque. *Sci. Rep.* **5**, 11823; doi: 10.1038/srep11823 (2015).

## Supplementary Material

Supplementary Information
