# Supplementary material for: Deterministic Domain Wall Motion Orthogonal To Current Flow Due To Spin Orbit Torque
Source: Sci Rep. 2015 Jul 3;5:11823. doi: 10.1038/srep11823 (PMC4490340; doi:10.1038/srep11823)
Supplement: Supplementary Information [file srep11823-s1.pdf]

Supplementary Material to accompany

"Deterministic Domain Wall Motion Orthogonal To Current Flow Due To Spin Orbit Torque"

Debanjan Bhowmik[1], Mark E. Nowakowski[1], Long You[1], OukJae Lee[1], David Keating[2], Mark Wong[1], Jeffrey Bokor[1,3] and Sayeef Salahuddin[1,3]

[1]*Department of Electrical Engineering and Computer Sciences, University of California Berkeley, Berkeley, CA 94720, USA*

[2]*Department of Physics, University of California Berkeley, Berkeley, CA 94720, USA*

[3]*Material Science Division, Lawrence Berkeley National Laboratory*

Section S1- Micromagnetic simulation showing that conventional/ bulk spin torque can move the transverse domain wall but not the longitudinal domain wall

When current flows through a ferromagnet, conduction electrons in the ferromagnet apply a "bulk spin torque" on the domain wall of the ferromagnet, given by $b_J\left(\vec{J_C}.\nabla\right)\vec{m} - \beta b_J\vec{m}\times(\left(\vec{J_C}.\nabla\right)\vec{m})$, where $b_J = \frac{\mu_B P}{eM_s}J_c$; μ_B- Bohr magnetron, P- spin polarization , e- charge of an electron, M_s- saturation magnetization of the ferromagnet, β- non-adiabatic parameter and J_c- charge current[1-4].

For a transverse domain wall, since the current flows across the domain wall, the magnetization has a non-zero gradient along the direction of the charge current ($\left(\vec{J_C}.\nabla\right)\vec{m} \neq 0$). So the bulk spin torque is non-zero. We perform micromagnetic simulations on Object Oriented MicroMagnetic Framework (OOMMF)[5] using the extension module for current induced domain wall motion (class spinTEevolve)[6] to show that a transverse domain wall moves due to the bulk spin torque when current flows across it. Only bulk spin torque and no spin orbit torque is present in this simulation (Fig. S1(a)).

A 600 nm long, 200 nm wide and 1 nm thick magnet is used for simulations, with a mesh size of 2 nm laterally and 1 nm across the thickness. For all micromagnetic simulation figures in the main paper and the supplementary material, blue dots with red background represent moments pointing out of the plane (-z) while red dots with blue background represent moments pointing into the plane (+z). The simulation parameters are: saturation magnetization M_s= 8 x 10^5 A/m (measured for our materials stack by performing Vibrating Sample Magnetometry), exchange constant A= 3 x 10^{-11} J/m, perpendicular anisotropy constant K= 6 x 10^5 J/m^3, spin polarization P=0.5, non-adiabatic parameter β=0.04 and charge current J_c= 10^8 A/cm^2. Starting from a transverse domain wall at the centre of the magnet, we see that the domain wall moves with time under the application of current due to the bulk spin torque (Fig. S1(a)). The domain wall moves in the direction of propagation of electrons, i.e., opposite to the direction of the current, as expected from theory[1-3].

When we simulate a longitudinal domain wall instead, current flows along the domain wall. In this case the direction, in which the gradient of the magnetization is not zero, and direction of the current are orthogonal, so $(\vec{J_C}.\nabla)\vec{m} = 0$. So the longitudinal domain wall does not experience a bulk spin torque and hence it does not move under the application of a current pulse, as we verify through micromagnetic simulations using the same simulation parameters as the transverse domain wall case (Figure S1(b)). Only bulk spin torque and no spin orbit torque is present in this simulation unlike the simulations of Figure 4 of the main paper.

In our experiments we move the longitudinal domain wall with current pulses in the presence of an in-plane magnetic field along the current direction (Figure 3 and Figure 4 of the main text). In Figure S1(c), we show simulations of a bulk spin torque acting on a longitudinal domain wall just like Fig. S1(b), with an in-plane magnetic field of 10G applied in addition to it along the current direction. We see that even in this case the longitudinal domain wall does not move with time. Fig. S1(d) shows that when the magnetic field is orthogonal to the current, the bulk spin torque does not move the domain wall either. Again, no spin orbit torque is considered in these simulations. Thus

we conclude that the bulk spin torque cannot move the longitudinal domain wall even in the presence of the magnetic field, and spin orbit torque is needed to explain our experimental data on longitudinal domain wall with current pulses.

S1(a)

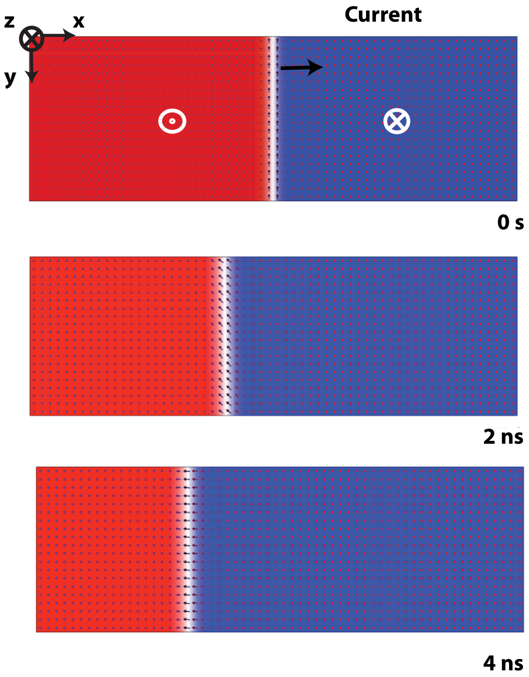

FIG. S1(a) Current flows in the +x direction, i.e. electrons move in the -x direction to apply the bulk spin torque on the transverse domain wall. Blue dots with red background represent moments pointing out of the plane (-z) while red dots with blue background represent moments pointing into the plane (+z). We see that the transverse domain wall moves along the flow of electrons with time as expected.

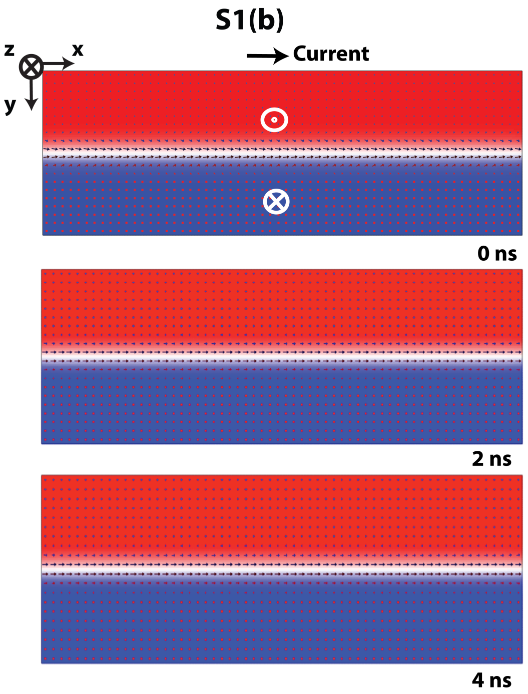

FIG. S1(b) Current flows in +x direction, i.e. electrons move in -x direction, but it cannot apply a bulk spin torque on the longitudinal domain wall. No magnetic field is applied. We see that the longitudinal domain wall does not move with time.

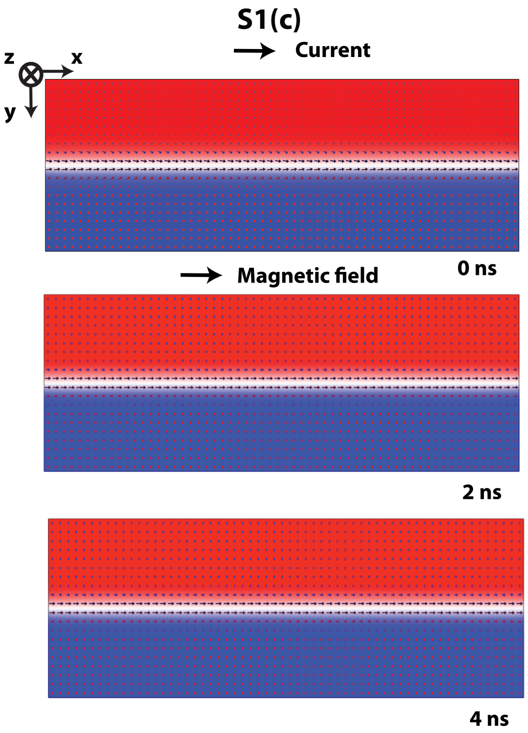

FIG. S1(c) Current flows in +x direction, i.e. electrons move in -x direction. An in-plane magnetic field of 10 G is applied in +x direction. We see that the longitudinal domain wall does not move with time.

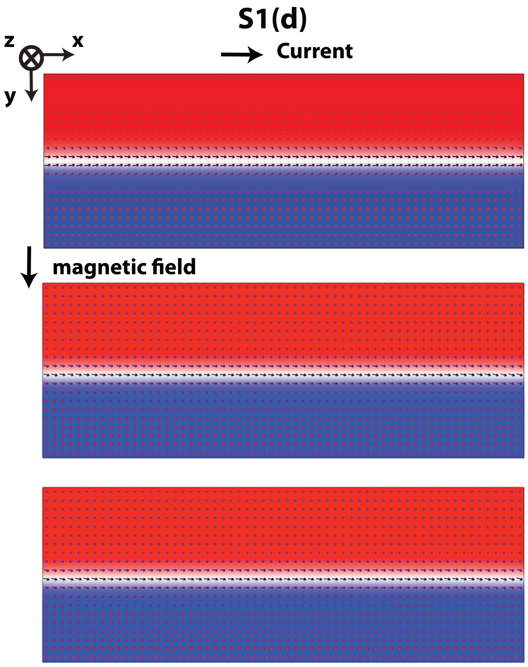

FIG. S1(d) Current flows in +x direction, i.e. electrons move in -x direction to apply the bulk spin torque on the longitudinal domain wall. An in-plane magnetic field of 10 G is applied in +y direction. We see that the longitudinal domain wall does not move with time.

Section S2- Characterization of the materials stack

A thin film stack of Ta (10 nm)/ CoFeB (1 nm)/MgO (1 nm)/ Ta (2 nm) has been sputter deposited on thermally oxidized Si substrate at room temperature. Vibrating Sample Magnetometry is used to characterize the magnetic properties of the thin film stack. When magnetic field is applied out of the plane, the magnet switches sharply close to 0 gauss with the remanent moment close to the saturation moment (Fig. S2). This suggests that out of the plane direction is the easy axis of the magnet. When the magnetic field is applied in plane, a hard axis plot is obtained for magnetic moment versus magnetic field

with a field of ~1500 G needed to saturate the magnet in the in-plane direction. Thus we confirm perpendicular magnetic anisotropy (P.M.A.) in our stack. The saturation magnetic moment is ~20 μ e.m.u measured for a 5 mm by 5 mm thin film sample. Thus the saturation magnetization of the 1 nm thick CoFeB layer is ~ 800 e.m.u./c.c. , or ~8×10^5 A/m. This value of saturation magnetization (M_s) is used in the micromagnetic simulations.

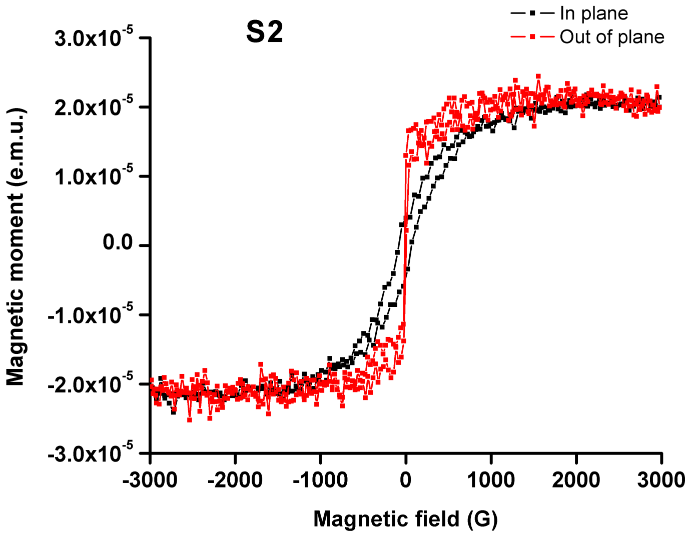

FIG. S2- Magnetic moment versus magnetic field plots for the out of plane direction (red plot) and the in plane direction (black plot) show that the easy axis is in the out of plane direction.

Section S3- Micromagnetic simulaton showing the formation of the domain wall at the centre of the bar due to current pulse

In Fig. S3(a), using Comsol simulations, we plot the Oersted field generated by the 7.5 x 10^6 A/cm^2 current pulse, which is needed to create the mixed state. We see that the edges experience ~10 G of magnetic field (red and blue patches in Fig. S3(a)) whereas the out of plane component of the magnetic field is negligible anywhere other than the very edges of the bar. The edges also have more defects than the centre due to fabrication issues and hence the magnetic material at the edges has lower perpendicular

magnetic anisotropy. Hence reverse domains nucleate at the edges of the bar when a current pulse of magnitude 7.5 x 10^6 A/cm^2 or higher is applied on the bar at a zero magnetic field. Also the Joule heating due to the fairly high magnitude of current pulse can help in the nucleation process.

Next we perform micromagnetic simulations to show how the domain wall moves from the edge to the centre of the bar once reverse domains nucleate at the edge of the bar. In Figure S3(b) the magnet is initially saturated "into the plane" (m_z=1; red dots in blue background). As current flows in +x direction, Oersted field generated by the current switches the moments near the upper edge of the bar (y<0) to "out of the plane" (m_z=-1, blue dots in red background). A longitudinal domain wall is formed as a result near the upper edge of the bar as shown in Fig. S3(b). We simulate a 600 nm long and 200 nm wide magnetic bar using the same simulation parameters as used in Supplementary Section 1 with the initial condition of a longitudinal domain wall near the upper edge of the bar (Fig. S3(b)-image of the magnet at time = 0 ns). We use the standard time evolver provided by OOMMF (Oxs_EulerEvolve) to let the system evolve with time taking into account all the energy terms- exchange energy, anisotropy energy and magnetostatic energy. We observe that the longitudinal domain wall moves in +y direction towards the centre with time to reduce the total energy of the system. This is because magnetostatic energy is minimum when the domain wall is at the centre of the bar. The final steady state of the system consists of a longitudinal domain wall at the centre (y=0) with the upper part of the bar (y<0) being polarized "out of the plane" and the lower part (y>0) being polarized "into the plane". The polarity of the domains matches with what we observe in the experiment (Figure 2(c) of the main paper).

Similarly starting from a magnet saturated in the "into the plane" direction current flowing in the –x direction nucleates "out of the plane" polarized domains at the lower edge of the magnet resulting in the formation of a longitudinal domain wall near the lower edge (Supplementary Fig. S3(c)- image of the magnet at time= 0 ns). As time progresses the longitudinal domain wall moves towards the centre and at the steady state we get a "mixed state" with equal area of "out of the plane" and "into the plane"

polarized domains. The polarity of the domains matches with what we observe in the experiment (Figure 2(c) of the main paper).

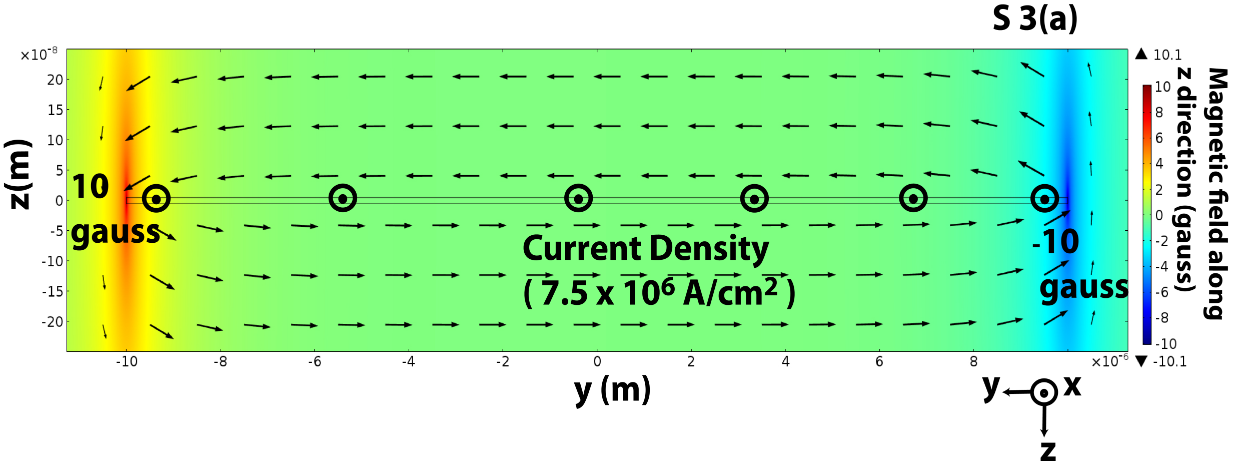

FIG. S3(a) Current flowing along the bar in Figure 2(c) of the main paper is shown to flow out of plane here (⊙). The same coordinate axes of Fig.S3a and S3b are used. x-axis represents the longitudinal direction of the bar, y-axis the transverse direction (width of Ta wire- 20 microns) and z axis the thickness (thickness of Ta wire- 10 nm). We see that the Oersted field is in the plane (y axis) near the centre of the bar but it is in the out of the plane direction (+z/-z) near the edge of the bar (red and blue patches). The estimated Oersted field for 7.5×10^6 A/cm^2 of current, used in the simulation, is ~ 10 G near the edge of the bar, as read from the colour bar for out of plane component of the magnetic field.

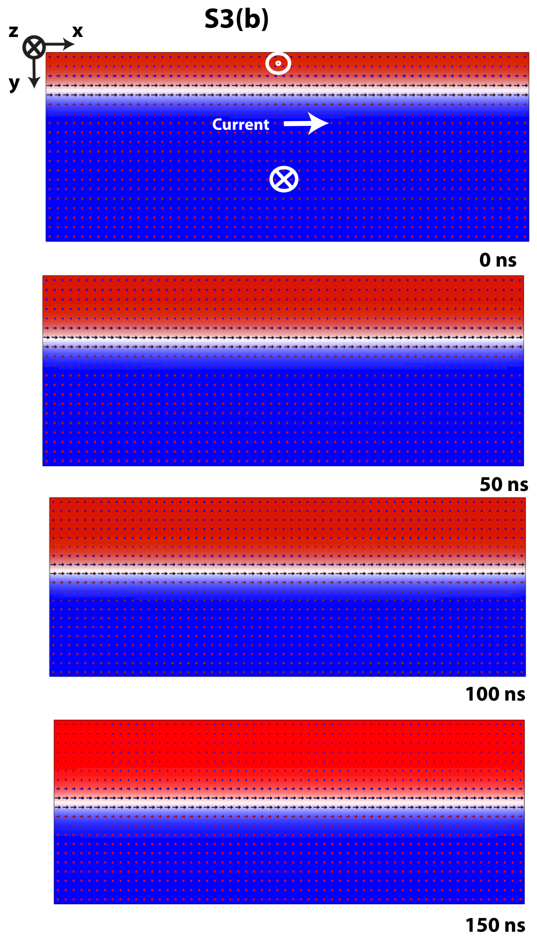

FIG S3(b)- Current in +x direction creates a domain wall at the upper edge of the bar ($y<0$) because of its Oersted field. Micromagnetic simulation shows that starting from the upper edge of the bar the domain wall moves to the centre of the bar at a zero magnetic field to lower its magnetostatic energy.

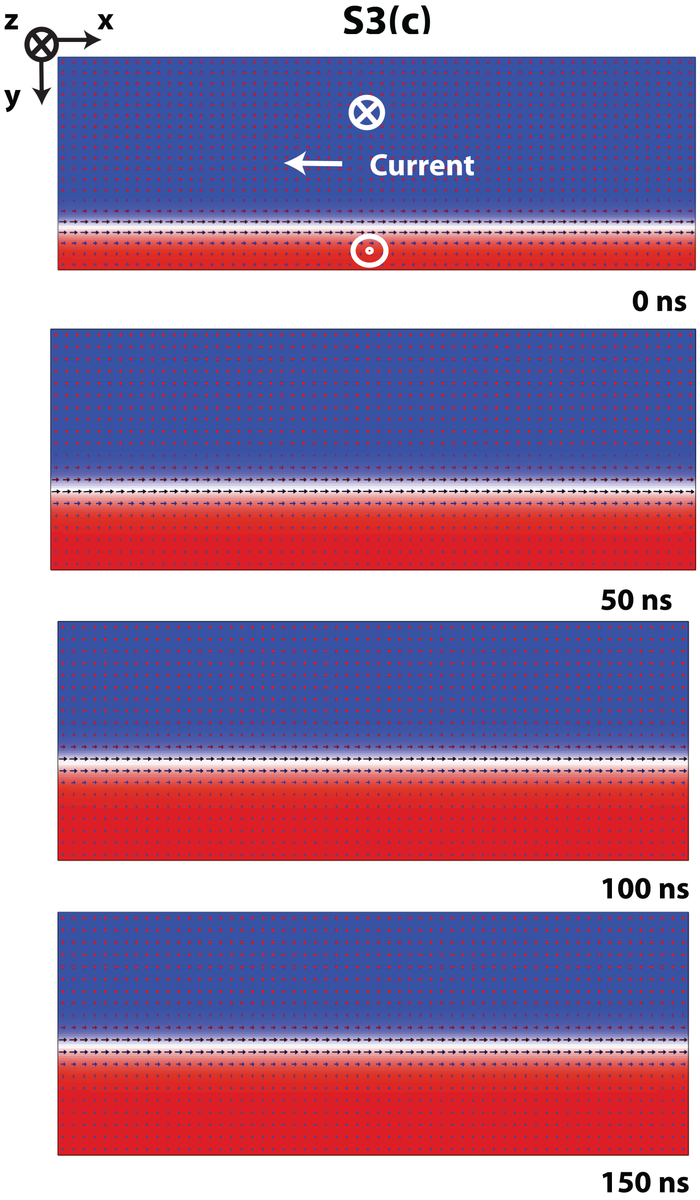

FIG S3(c)- Current in -x direction creates a domain wall at the lower edge of the bar (y>0) because of its Oersted field. Micromagnetic simulation shows that starting from the lower edge of the bar the domain wall moves to the centre of the bar at a zero magnetic field to lower its magnetostatic energy.

Section S4- Domain wall motion for different current pulses and in-plane magnetic fields

In Figure 3a of the main manuscript, we present MOKE images that show how the longitudinal domain wall moves under two different cases: magnetic field in –x and current in +x; and magnetic field in +x and current in +x. Here we show MOKE images for the third and fourth case: magnetic field in +x and current in –x [Figure S4(a)]; and . magnetic field in -x and current in –x. When magnetic field is in –x direction and current is in –x direction, the domain wall moves in +y direction and final state of the magnet is "out of the plane" (-z) while when magnetic field is in +x direction and current is in –x direction, the domain wall moves in –y direction and final state of the magnet is "into the plane" (+z). The final state of the magnet in each case is consistent with the R_{AHE} measurement (contour plot) in Fig. 3b of the main manuscript.

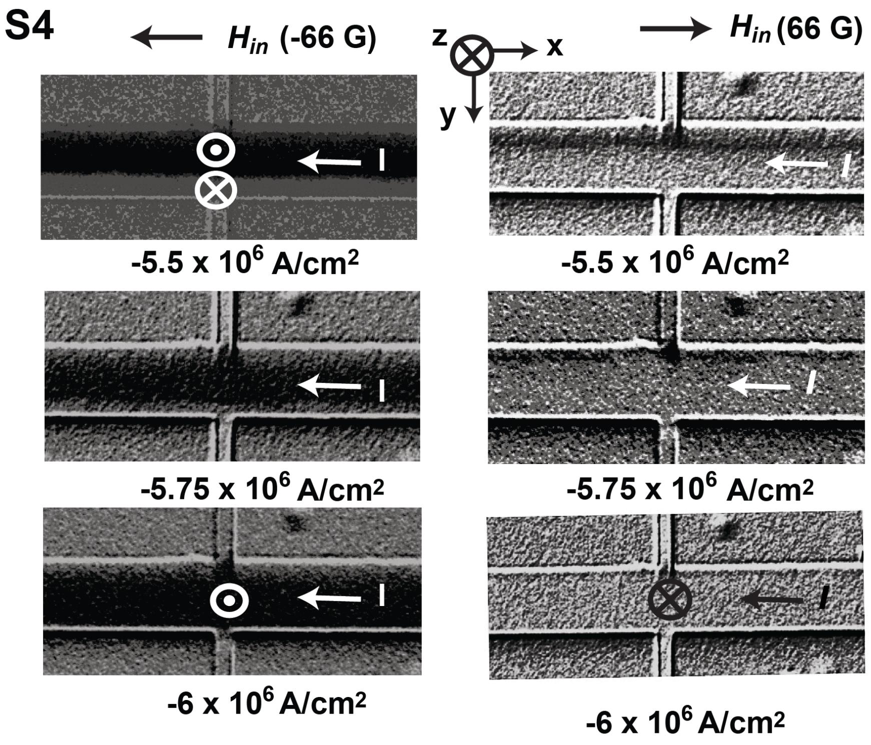

FIG S4. MOKE images showing domain wall motion when current is in –x direction and magnetic field is applied in –x and +x direction.

Section S5- Negligible Dzyaloshinskii-Moriya Interaction (DMI) in our sample

To measure the magnitude of DMI in our sample we create a transverse magnetic domain wall in our magnetic bars. The R_{AHE} versus out of plane magnetic field plot of Figure 2a (main text) shows that the magnet can be switched by 30 G of magnetic field. However the R_{AHE} measured is only proportional to the average moment in the intersection region of the two Hall bars. MOKE imaging of the switching process shows that a transverse domain wall is nucleated near an end of the bar when an out of plane switching field of magnitude less than 30 G is applied. As the field increases this wall moves along the bar and switches the whole magnet. Such a transverse domain wall is shown in Figure S5(a).

If the DMI of the system is considerable and hence the transverse domain wall is a Neel wall, application of a current pulse along the bar will move it even in the absence of an external magnetic field[4]. So we saturate the magnetic bar in the "into the plane" direction and apply a magnetic field in out of the plane direction, which is less than 30 G. A transverse domain wall nucleates near the right end of the bar every time (Figure S5(a) and S5(b)). Then the magnetic field is turned off. The bar is imaged to record the position of the transverse wall. Then a current pulse is applied along the bar at a zero external magnetic field. The bar is imaged at the end of the current pulse and the position of the domain wall after the current pulse is recorded (Fig. S5). We repeat this experiment several times changing the magnitude and polarity of the current pulse. We observe that the position of the transverse domain wall does not change before and after the current pulse. The domain wall gets distorted after a current pulse of higher magnitude but its average position does not change. The distortion is related to the onset of formation of the "mixed state" because we are applying a current pulse along the bar at a zero magnetic field just like in Figure 2 of the main paper.

From the experimental result that a current pulse across a transverse domain wall does not move it unlike the experiments reported by S. Emori and colleagues[4], we infer that the DMI in our system is negligible.

S5(a)

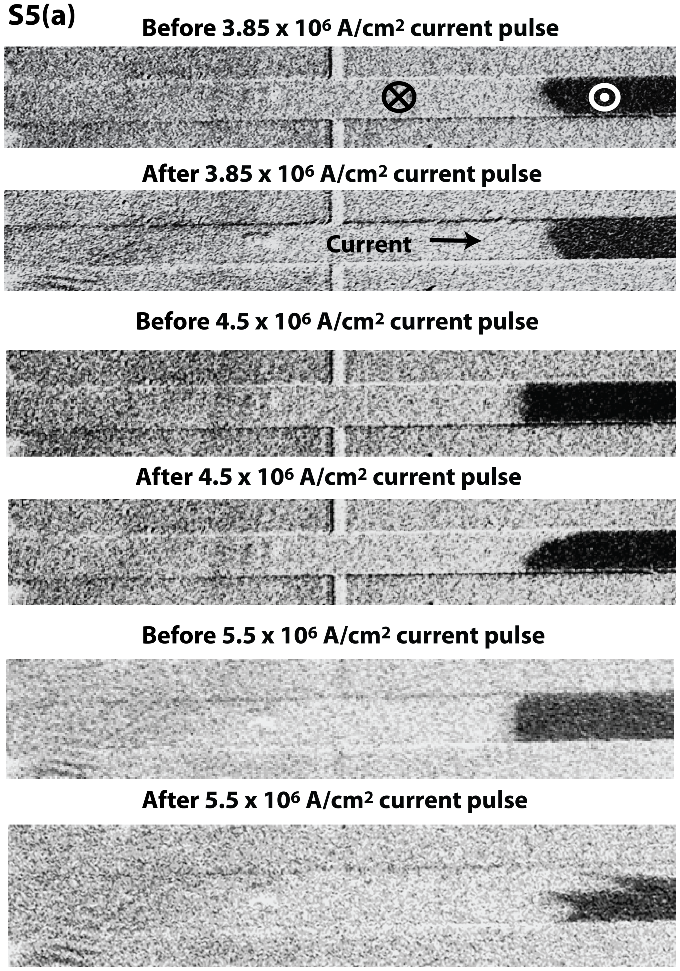
Before 3.85 x 10⁶ A/cm² current pulse
After 3.85 x 10⁶ A/cm² current pulse
Current
Before 4.5 x 10⁶ A/cm² current pulse
After 4.5 x 10⁶ A/cm² current pulse
Before 5.5 x 10⁶ A/cm² current pulse
After 5.5 x 10⁶ A/cm² current pulse

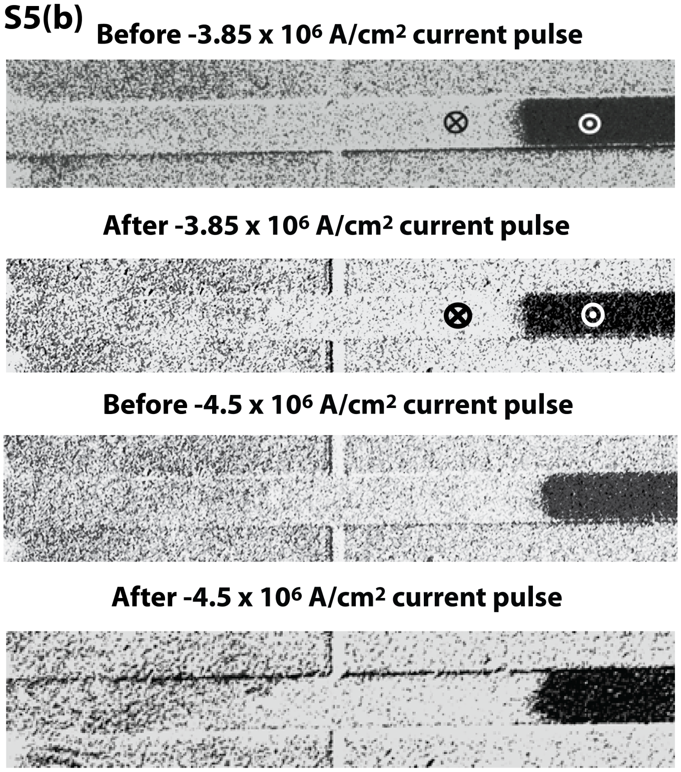

FIG 5a,b MOKE images of the transverse domain wall before and after the application of current pulses of different magnitudes and polarities show no movement of the transverse domain wall due to the current pulses.

Section S6- Evidence of the domain wall motion not being a result of Oersted field of the current or field like spin orbit torque

We have demonstrated experimentally that a longitudinal domain wall can be moved from the centre to the edge of the bar with a current pulse along the bar (Fig. 3). Since the direction of motion of the domain wall is dependent on the polarity of the current, it cannot be attributed to Joule heating.

When a current pulse is applied on the longitudinal domain wall, which is at the centre of the bar, the domain wall experiences the Oersted field, generated by the current, which acts in the in-plane direction along the width of the bar (Figure S3c of Supplementary Information). The Oersted field generated by the current pulse or the field like spin orbit

torque from the current cannot explain this kind of domain wall motion as well. The Oersted field or field like torque (Rashba field), if applied along with the external magnetic field, can change the configuration of the moments in the domain wall but cannot move the wall. We performed micromagnetic simulation (Fig. S10) where a field of 10 G is applied in x direction, similar to the external magnetic field in the experiment, and a field of 2.5 G in y direction, which can represent the Oersted field or the field like spin orbit torque from the current pulse. We observe that the domain wall does not move with time under the influence of these two orthogonal fields.

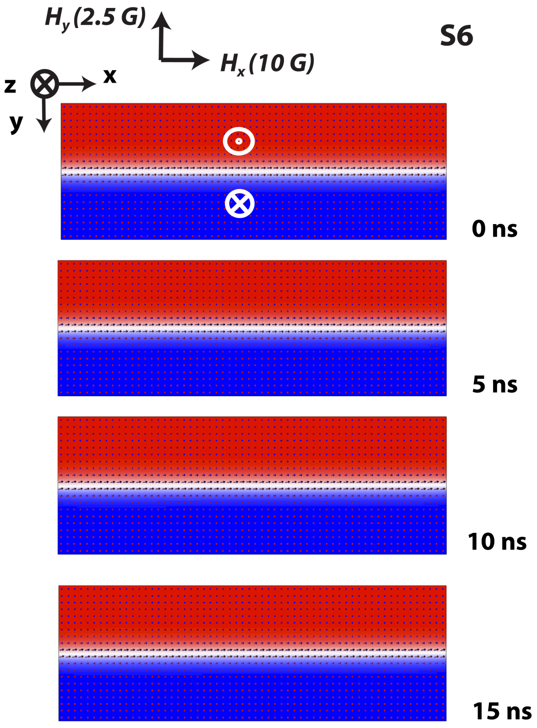

FIG S6- Micromagnetic simulation of time evolution of a longitudinal domain wall under the application of 10 G along x and 2.5 G along y shows no domain wall motion.

Section S7- Calculation of the spin orbit torque efficiency

In Fig. 3(c) of the main text we observe that the R_{AHE} increases with the magnitude of the current pulse for a given in-plane magnetic field because a stronger current pulse moves the domain wall further (Fig. 3(a) of main paper). The rate of this increase is low for

small magnetic fields but increases with increasing magnitude of field (Fig. S7(a)). For a magnetic field of magnitude 45 G and above (we use negative sign to represent magnetic field in –x direction, so we talk about magnitudes to imply the strength of the field) R_{AHE} increases with the current density of the pulse till it reaches saturation. This corresponds to the longitudinal domain wall moving all the way from the centre to the edge of the bar to switch the magnet from "mixed" state to a saturated "into the plane" state (Fig. 3). Using linear fit around the region of the curves where R_{AHE} increases with current, represented by dotted straight lines in Fig. S7(a), we obtain the rate of increase of R_{AHE} with current ($\frac{\partial R_{AHE}}{\partial J_C}$). In Fig. 7(b) we plot $\frac{\partial R_{AHE}}{\partial J_C}$ against the applied in-plane magnetic field. We see that $\frac{\partial R_{AHE}}{\partial J_C}$ increases with the magnitude of applied magnetic field till it reaches saturation for 45 G and above. This happens because a magnetic field of sufficient magnitude (45 G and above in this case) drives the net moment of the domain wall completely in its direction. Hence the "effective out of plane magnetic field" experienced by the domain wall, which is the cross product of net moment in the domain wall and the spin polarization, is the maximum. Stronger the effective field, the higher is the domain wall motion due to the current and higher is the $\frac{\partial R_{AHE}}{\partial J_C}$. We take average of $\frac{\partial R_{AHE}}{\partial J_C}$ values for magnetic fields of magnitude 45 G and above to get a value of

$7.39 \times 10^{-7}\ \Omega/(\mathrm{A/cm^2})$.

We have also shown that the longitudinal domain wall can be moved from the centre of the bar to the edge with external magnetic field, applied in the out of the plane direction (Fig. 2 of main text). Comparing the change in R_{AHE} corresponding to the domain wall motion due to the externally applied out of plane field with the change in R_{AHE} due to the current pulse would give us an estimate of the "effective out of plane magnetic field" experienced by the domain wall due to the current. Fig. S7(c) shows that R_{AHE} increases with an external magnetic field in the out of the plane direction (H_{out}) corresponding to the domain wall motion of Fig. 2b. R_{AHE} finally reaches saturation because the domain wall has moved all the way from the centre to the edge. $\frac{\partial R_{AHE}}{\partial H_{out}}$ corresponding to the linear region of the curve is 0.15 Ω/G. Thus, "effective out of

plane field" experienced by the domain wall due to the current $\frac{\partial H_{out}}{\partial J_C} = (\frac{\partial R_{AHE}}{\partial J_C})/(\frac{\partial R_{AHE}}{\partial H_{out}})$= 4.92×10^{-6} G/(A/cm^2).

Using the 1D domain wall theory[7], Thiaville and colleagues[8] developed an expression for the "effective out of plane" spin orbit field experienced by a transverse Neel wall when current flows across it. The spin polarization at the interface is orthogonal to the net magnetic moment of the Neel wall and applies an effective out of plane field $H_{out} = \frac{\pi}{2}\frac{\hbar\theta}{2et_F M_s}$, on the domain wall, where θ is the spin orbit torque efficiency, e is the charge of an electron, $\hbar$ is the Planck constant, μ_0 is the vacuum permeability, M_s is the saturated magnetization of the ferromagnet (8×10^5 A/m, as measured for our thin film stack by Vibrating Sample Magnetometry) and t_F is the thickness of the ferromagnet (1 nm in our case). The longitudinal domain wall in our experiment is a Bloch wall and the net magnetic moment of the wall is along the direction of the applied in-plane field. Since current flows along the longitudinal wall, the spin polarization ($\vec{\sigma}$) at the interface due to SHE is orthogonal to the net magnetic moment of the domain wall (black arrow of Fig. 4) as shown in Fig. 4a and Fig. 4b of the main paper. So the same expression for the "effective out of plane field" can be used for our experiment. The experimental value of $\frac{\partial H_{out}}{\partial J_C}$ is used in the expression to extract the spin Hall angle (θ_{SHE}), which turns out to be equal to 0.076.

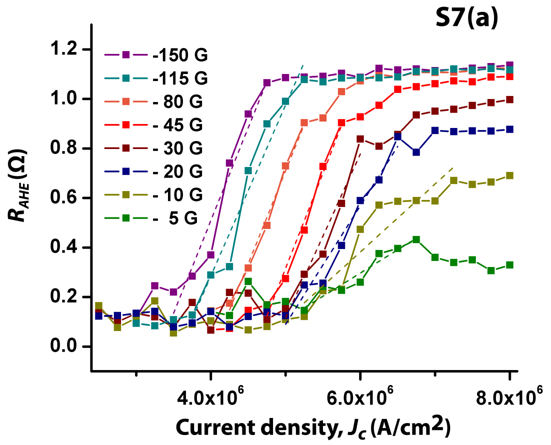

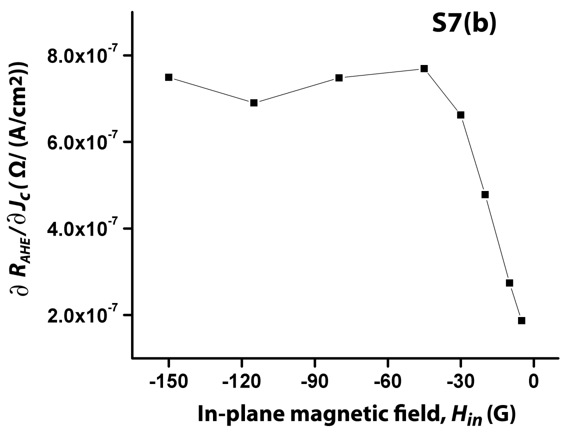

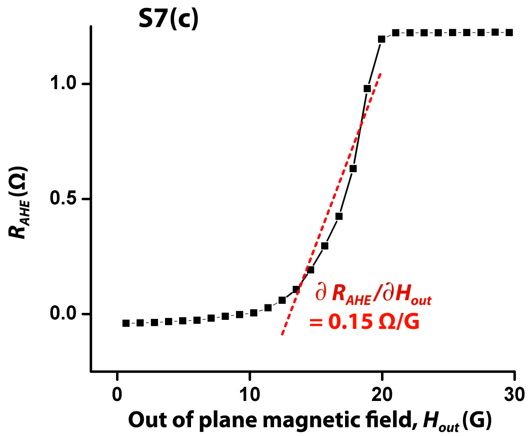

FIG 7(a) Starting from a longitudinal domain wall at the centre of the bar every time, current pulses of different magnitude are applied for an applied in-plane magnetic field in –x direction and R_{AHE} is measured after every pulse. R_{AHE} is plotted against the current density of the current pulses for different in-plane fields. Using linear fit for the region where R_{AHE} increases with the current, $\frac{\partial R_{AHE}}{\partial J_C}$ values (slopes of the dotted lines) for different in-plane fields are obtained. (b) Plot of $\frac{\partial R_{AHE}}{\partial J_C}$, obtained in Fig. 10a versus applied in-plane field. (c) Starting from a longitudinal wall at the centre of the bar, out of plane magnetic field (H_{out}) is applied of increasing magnitude. The R_{AHE} increases with increasing field corresponding to the domain wall moving from the centre of the bar to edge to switch the magnet from "mixed" state to saturated "into the plane" state (Fig. 2a).

Using a linear fit for the region where R_{AHE} increases with H_{out} , $\frac{\partial R_{AHE}}{\partial H_{out}}$ (slope of the dotted line) is obtained.

Section S8- Transverse magnetic field does not move the longitudinal domain wall

A longitudinal domain wall is created at the centre of the rectangular bar by a current pulse of magnitude 7.5 x 10^6 A/cm^2 and duration 1s following the same procedure as every other time in this work (Fig. 1(d) and Fig. 2(a) of main manuscript). Once the domain wall is formed, transverse in-plane fields of magnitudes 100 G and 500 G are applied in the absence of any current. Position of the domain wall does not change (Fig. S8).

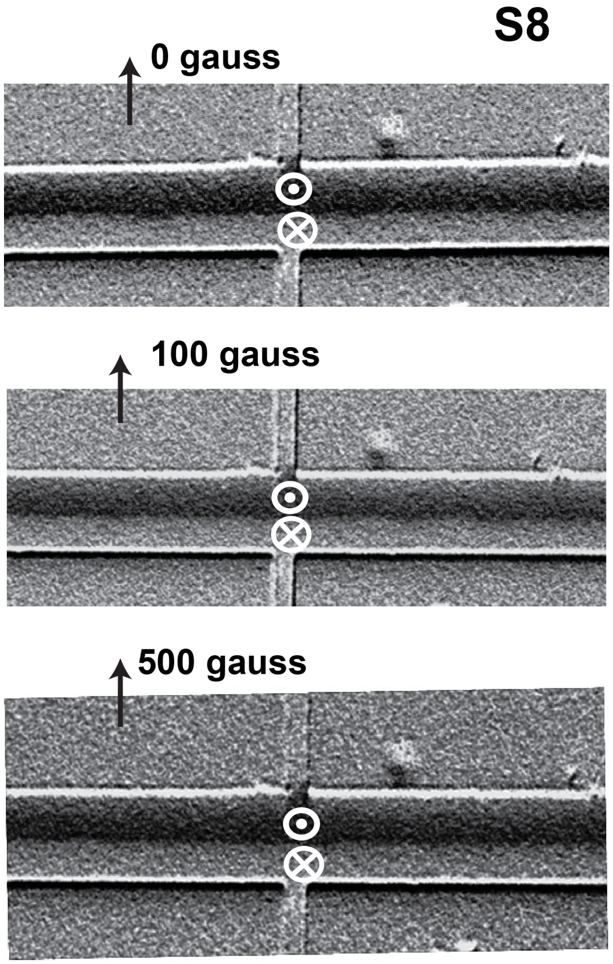

Fig. S8- A longitudinal domain wall, created at the centre of the bar, does not move when 100 G or 500 G of magnetic field is applied on it.

Section S9- Magnetic switching from saturated state under the application of an in-plane magnetic field and current

By applying current through the same Hall bars of Figure 1 of main manuscript in the presence of in-plane magnetic field and measuring the anomalous Hall resistance R_{AHE}, we observe the dependence of magnetization on the polarities of the applied current and in-plane magnetic field. We observe a hysteric switching of magnetization with current when 50 gauss of magnetic field is applied in +x direction (Fig. S9a). +x direction is shown in Fig. 3 of main manuscript. Positive polarity of current (+x, with respect to Figure 3 of main manuscript) beyond a certain magnitude (~ 5×10^6 A/cm^2) switches the magnet out of the plane, which corresponds to R_{AHE}= -1 (Fig. 3 of main manuscript), while negative polarity of current beyond that magnitude switches the magnet into the plane, which corresponds to R_{AHE}= +1 (Fig. 3 of main manuscript). On the other hand when 50 gauss of magnetic field is applied in –x direction, +x current switches the magnet into the plane (R_{AHE}= +1) while –x current switches the magnet out of the plane (R_{AHE}=-1) (Fig. 9b). Thus, when current and magnetic field are in the same direction the final state of the magnet is in the out of plane direction. When current and magnetic field are in the opposite direction the final state of the magnet is in the into the plane direction. Basically, the final state of the magnet is the cross product of the applied magnetic field (H_{in}) and the spin accumulation ($\vec{\sigma}$) at the Ta-CoFeB interface, which is orthogonal to the current flow direction as shown in Fig. 1 and Fig. 4 of the main manuscript. Final state = $\overrightarrow{H_{in}} \times\vec{\sigma}$. The final state of the magnet, observed in this switching experiment, is consistent with the switching experiment performed by Liu *et al.* on Ta/CoFeB/MgO stack, exhibiting perpendicular anisotropy[9]. It is to be noted that the handedness of Fig. S9 here and Figure 2 of Ref. 9 (Liu *et al.)* look opposite but that is because we choose positive R_{AHE} for "into the plane" state and negative R_{AHE} for "out of the plane" state as our convention throughout our paper while Liu *et al.* choose positive R_{AHE} for "out of the plane" state and negative R_{AHE} for "into the plane" state.

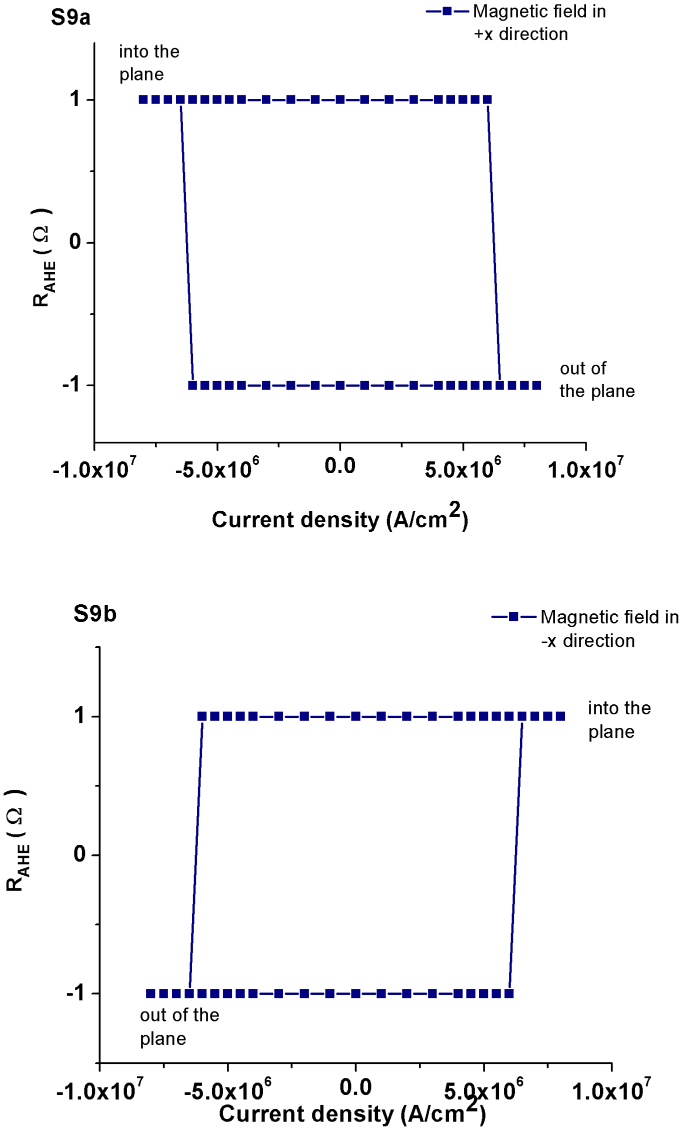

Figure S9- Positive current refers to current applied in +x direction and negative current refers to current applied in –x direction (+x direction shown in Fig. 3 of main paper). When current and magnetic field are in the same direction (current I in +x direction with in-plane magnetic field H_{in} in +x direction OR current I in -x direction with in-plane magnetic field H_{in} in -x direction) the final state of the magnet is in the "out of plane" direction (R_{AHE}=-1). When current and magnetic field are in the opposite direction (current I in +x direction with in-plane magnetic field H_{in} in -x direction OR current I in -x direction with in-plane magnetic field H_{in} in +x direction) final state of the magnet is in the "into the plane" direction (R_{AHE}=1).

Section S10- Measurement of Joule heating due to current

We have measured the resistance of a 20 micron wide magnetic bar made from the same stack of Si (substrate)/ SiO_2 (100 nm)/ Ta (10 nm)/ CoFeB (1 nm)/ MgO (1 nm)/ Ta (2 nm). Four probe measurement technique is used (Fig. S10a). The resistance measured at room temperature is 300 Ohm. Temperature of the sample is first increased using a heater. Change in resistance is plotted as a function of temperature in Fig. S10b. We see that the resistance goes down by ~6.5 Ohm as the temperature increases from room temperature (~290K) to 400K. Decrease of resistance of Ta with increase in temperature in this temperature range has been reported by Schwartz *et al.*[10]

Change in resistance is next plotted against dc current density through the device (Fig. S10c). We do four-probe measurement on the same device to measure a resistance as a function of the current through the device. Since we used 1 s current pulses for the domain wall motion reported in the paper, we need to account for the heating due to the current over the period of 1s. As a result, to obtain the plot of resistance versus current density, we first measure the resistance with a small current (5 x 10^4 A/cm^2). We call this the base resistance. Then high current of a particular magnitude is applied continuously for 1s and the resistance is measured at the end of 1 s with that current still on. It is then subtracted from the base resistance to get the change in resistance, corresponding to that current magnitude. The same experiment is repeated for several high current values and the change is resistance is plotted as a function of the corresponding magnitudes of current density.

We see that as the current increases, resistance goes down. This is because as more current flows temperature of the device goes up due to Joule heating and resistance drops because of increase in temperature. The maximum current density used in our experiment to move the longitudinal domain wall from the center to the edge of the magnet (Figure 3 of main manuscript) is 7×10^6 A/cm^2. From the plots in Fig. S10, we infer that for such current density the temperature of the device can increase up to ~400 K (~127^0 C).

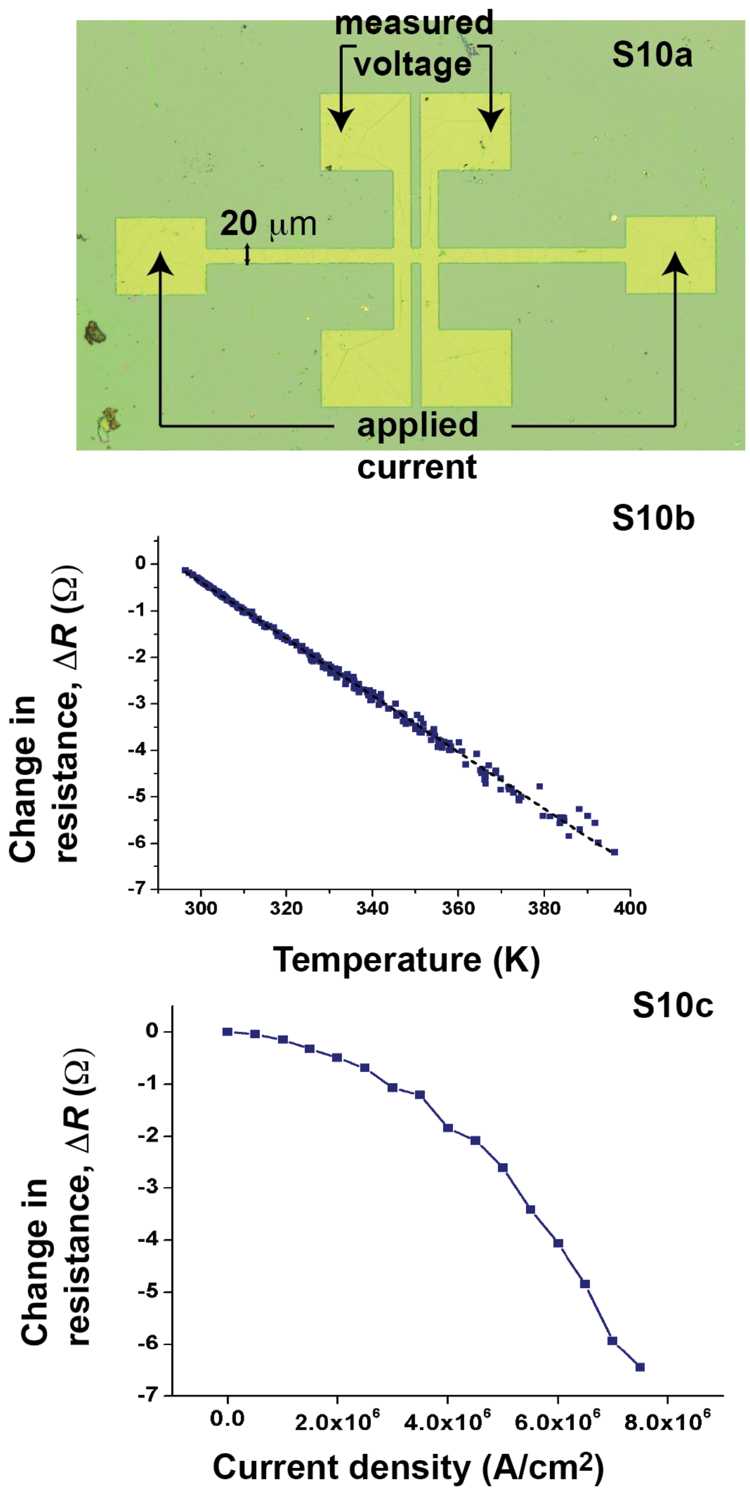

Figure S10- a, Double Hall bar fabricated for four probe measurement of resistance b, Change in resistance from resistance at room temperature plotted as a function of

increase in temperature. c, Change in resistance is plotted as a function of increase in dc current density.

References

1. Zhang, S. & Li, Z. *et al.* Roles of non-equilibrium conduction electrons on the magnetization dynamics of ferromagnets. *Phys. Rev. Lett.* **93,** 127204 (2004).

2. Thiaville, A., Nakatani, Y., Miltat, J. & Suzuki, Y. Micromagnetic understanding of current-driven domain wall motion in patterned nanowires. *Europhys. Lett.* **69,** 990-996 (2005).

3. Tatara, G. & Kohno, H. Theory of current driven domain wall motion: Spin transfer versus momentum transfer. *Phys. Rev. Lett.* **92,** 086601 (2004).

4. Emori, S., Bauer, U., Ahn, S., Martinez, E. & Beach, G. S. D. Current driven dynamics of chiral ferromagnetic domain walls. *Nature Mater.* **12,** 611-616 (2013).

5. Donahue, M. J. & Porter, *D. G. OOMMF User's Guide, Version 1.0, Interagency Report NISTIR* 6376 (1999).

6. OOMMF extension module for current induced domain wall motion- IBM Research- Zurich.

7. Malozemoff, A. P. & Slonczewski, J. C. *Magnetic Domain Walls in Bubble Material* (Academic, 1979).

8. Thiaville, A., Rohart, S., Jué, É., Cros. V. & Fert, A. Dynamics of Dzyalonshinskii domain walls in ultrathin magnetic films. *Europhys. Lett.* **100,** 57002 (2012).

9. Liu, L. *et al.* Spin-torque switching with the giant spin Hall effect of tantalum. *Science* **336,** 555-558 (2012).

10. Schwartz, N., Reed, W. A., Polash, P. and Read, M. H. Temperature coefficient of resistance of beta-Tantalum films and mixtures with b.c.c. Tantalum. *Thin Solid Films.* **14** (2), 333-346 (1972).
